# Characterisation of the function of a lncRNA containing SINE-VNTR-Alu 67 to regulate the genes at the *MAPT* locus

**DOI:** 10.3389/ebm.2025.10805

**Published:** 2025-11-27

**Authors:** Kynen Piacentini, Alexander Fröhlich, Abigail Pfaff, Sulev Kõks

**Affiliations:** 1 Perron Institute for Neurological and Translational Science, Perth, WA, Australia; 2 Centre for Molecular Medicine and Innovative Therapeutics, Murdoch University, Perth, WA, Australia; 3 UK Dementia Research Institute, University of Edinburgh, Edinburgh, United Kingdom

**Keywords:** SINE-VNTR-Alu 67, LncRNA, MAPT locus, differential gene expression, RT-qPCR

## Abstract

Parkinson’s disease (PD) is a complex neurodegenerative disease that involves many interlinking pathways and genetic elements that remain to be fully understood and characterised. Non-coding genetic elements have long been overlooked, however recent advancements in the field have highlighted their importance with an area of interest being transposable elements. SINE-VNTR-Alu (SVA) elements are the youngest and smallest subset of retrotransposons that are only found within hominid species. SVAs have been shown to have strong regulatory impacts within our genome and can affect progression of neurodegenerative disease such as PD. Previous studies identified an SVA, polymorphic for its presence/absence, that was associated with changes in gene expression at the *MAPT* locus. This particular SVA is located within a long non-coding RNA (lncRNA) and is known as SVA_67. Here, we evaluated the SVA67-lncRNA effects on gene expression within the *MAPT* locus, a region associated with several neurodegenerative diseases in the SH-SY5Y cell line. The expression of SVA67-lncRNA in the SH-SY5Y cell line was associated with differential expression of several genes at the *MAPT* locus including *MAPT*, *KANSL1*, *ARL17A/B*, *LRRC37A/2*, and *NSF*. This study provides the first analysis of this SVA67-lncRNA and potential evidence for its involvement in complex diseases, such as PD.

## Impact statement

This work shows that the novel SVA67-lncRNA has regulatory function within a Parkinson’s disease associated locus and affects expression of genes previously shown to be linked to the same disease progression. This project also exposes a potential link between this SVA67-lncRNA and Parkinson’s disease progression when comparing results with other GWAS studies. The results of this study encourage future studies to incorporate functional assays to assess the effects on pathophysiological pathways. With the aetiology and genetics of Parkinson’s disease not fully understood, this study provides further information into the missing heritability.

## Introduction

Neurodegenerative diseases are the leading source of disability in the world, with Parkinson’s disease (PD) increasing the most in number of individuals affected [1, 2]. PD is a progressive neurodegenerative disorder caused by a complex interplay of genetic and environmental factors, which raises the difficulty in fully understanding the aetiology of PD. The current pathological hallmarks of PD include the degeneration of dopaminergic neurons in the substantia nigra and the formation of Lewy bodies through the misfolding and accumulation of alpha-synuclein (α-syn) [3]. Currently, there are three major interconnected pathophysiological pathways that are disrupted and hold a relationship with α-syn in the pathogenesis of PD. This includes disrupted function of the autophagy-lysosome, mitochondrial and vesicular pathway [4–6]. PD can be categorised into either familial PD, accounting for 15% of all PD patients, and sporadic PD where there is no family history of PD, accounting for the remaining 85% of patients [7]. There is great difficulty in understanding the genetic makeup of a sporadic PD case as there are currently over 200 PD-related genes that have been identified [8]. There are also many gene-gene interactions that can occur which further raises the complexity of this disease [9, 10].

There have been several large-scale genome-wide association studies (GWAS) identifying PD-associated risk signals [11, 12]. Nalls at al [11]. identified 90 PD-associated risk signals across the genome in a European population. Even though this study was the largest study at the time doubling the number of known PD risk variants, it still only explained 16–36% of heritable risk of PD. Most recently, a study by Kim et al. [12] expanded on this previous study by including individuals of European, East Asian, Latin American, and African ancestry identifying 78 independent genome-wide significant loci, which included 12 potentially novel loci. With the amount of genetic risk variants identified and the potential of large numbers of common genetic variants required to contribute to the risk of developing PD, known as polygenic inheritance, it becomes increasingly important to investigate the remaining large proportion of contributing genetic factors. One area that needs attention involves non-coding areas of the genome such as long non-coding RNAs (lncRNAs) and transposable elements (TEs).

Approximately 45% of the human genome is comprised of TEs [13]. They can be classified into either transposons or retrotransposons depending on their mechanism of movement. Only a few subsets of the latter remain active within the human genome today, that being the long interspersed nuclear element-1s (LINE-1), *Alu*, and SINE-VNTR-Alus (SVAs). These elements use a “copy and paste” mechanism to mobilise within the human genome creating new insertions and generating genetic diversity. SVAs are the both the youngest and least understood subset of active retrotransposons in which are only found within hominid species [14]. A study by Pfaff et al. [15] identified a retrotransposon insertion polymorphism (RIP) identified as SVA_67. Through bioinformatic and functional studies, SVA_67 has strong regulatory effects and can alter the expression of several genes within the *MAPT* locus, a locus that has been strongly associated with PD [16, 17]. The *MAPT* locus comprises two major haplotypes, H1 and H2. The H2 haplotype is defined by a large inversion polymorphism and notably lacks the SVA_67 element, which is present in the H1 haplotype [16]. The major haplotype, H1, has been genetically associated with increased risk for multiple neurodegenerative disorders, including PD [11, 18]. An SVA_67 insertion polymorphism has been identified within a lncRNA (GenBank reference BC011194), termed SVA67-lncRNA. The focus of this study is to provide preliminary evidence of transcriptional regulation potentially caused by this SVA67-lncRNA, acknowledging the need for future *in vivo* functional validation.

## Materials and methods

### Introduction of SVA67-lncRNA in SH-SY5Y cell line

**FIGURE 1:**
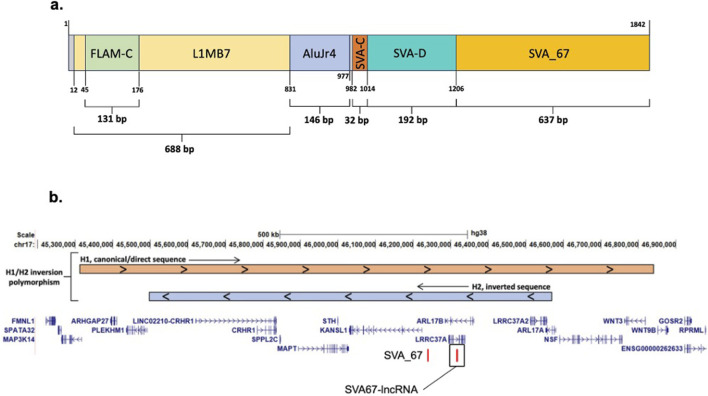
SVA67-IncRNA structure and schematic representation of the location of SVA_67. **(a)** Structure of entire SVA67-IncRNA cDNA Template, including the SVA_67 (IMAGE clone 4151959, GenBank reference BC011194). Structure of Plasmid template used for transfection in this study. Total length is 1842bp. **(b)** Schematic modified from UCSC genome browser hg38 and Frohlich et al. [16] of the MAPT locus on chromosome 17 showing the location of SVA_67 and the SVA67-IncRNA.

The SVA67-lncRNA containing plasmid was grown and isolated using *Escherichia coli* (*E. coli*) bacteria and a Midiprep Plasmid kit (Thermo Fisher) according to manufacturer’s instructions. The plasmid contained an engineered pCMV_SPORT6 plasmid backbone and the SVA67-lncRNA template (Horizon Discovery, IMAGE clone 4151959, GenBank reference BC011194). cDNA structure of the SVA67-lncRNA template can be found in Figure 1a. Plasmid extraction from *E.coli* was assessed by PCR using the primers found in Supplementary Table S1 in the following reaction and reagents (final concentration): Template DNA (10 ng/μL), KOD hot start buffer (1 × Merck), MgSO_4_ (1.5 mM, Merck), dNTPs (0.2 mM each), betaine (1 M, Sigma), Primers (0.75 ng/μL each, Sigma), KOD hot start DNA polymerase (0.02 U/µL), made up with nuclease free water to a final volume of 20 µL.

For transfection, 60,000 SH-SY5Y cells were provided by the Motor Neuron Disease group at the Perron Institute of Neurological and Translational Science from the American Type Culture Collection (ATCC). SH-SY5Y cells are widely used in neurodegeneration research. They were selected as a model system for this study due to their human neuronal origin, their ability to express the target genes analysed and their ease of transfection capabilities, allowing reliable detection of transcriptional changes. Cells were transfected with the SVA67-lncRNA plasmid in progressive increasing concentrations using Turbofect transfection reagent (Thermo Fisher) according to manufacturer’s instructions and incubated for forty-eight hours at standard growth conditions. To confirm transfection, a PCR was performed using the primers (synthesised by IDT Pty Ltd.) SVA_67_Fw and SVA_67_Rv, lncRNA_SVA_67_Fw1 and lncRNA_SVA_67_Rv1, lncRNA_SVA_67_Fw2 and lncRNA_SVA_67_Rv2 in the following reactions and reagents (final concentration): Template RNA (20 ng), 2x Reaction mix, Primers (10 µM each, IDT), Superscript III RT/platinum taq mix, made up with nuclease free water to a final volume of 12.5 µL. Complete details of primer sequences, expected product size and cycling conditions can be found in Supplementary Tables S1, S2.

### qPCR of target gene expression after the addition of SVA67-lncRNA

Quantification of target gene expression was performed using qPCR to assess the effects on expression in response to the introduction of various concentrations of SVA67-lncRNA plasmid. To generate cDNA from generated SH-SY5Y cell lines for qPCR experiments, total RNA was extracted using the PureLink RNA Mini Kit (Thermo Fisher). Extracted RNA then underwent DNase treatment using the TURBO DNA-free Kit (Thermo Fisher) and then the Superscript III Reverse Transcriptase Kit (Thermo Fisher) was used for first-strand cDNA synthesis according to manufacturer’s instructions.

Duplex TaqMan Gene Expression Assays were used for qPCR analysis under the following cycling conditions: 95 °C for 2 min, 95 °C for 20 s, 62 °C for 10 s and 70 °C for 50 s, repeating steps 2–4 39 more times. Two endogenous controls, *GAPDH* and *HPRT1*, were used to normalise the expression results and correct for any potential biases caused by RNA input, reverse transcription efficiency, or sample quality. Two biological replicates were analysed with four technical replicates for each gene at each concentration level.

A CFX Opus 384 real-time PCR system (BioRad) was used to analyse gene expression by collecting quantification cycle (Cq) values. Target gene expression was quantified using the 2-delta delta Ct method comparing samples to an empty vector sample. Significance was determined using a one sample t-test generating p-values at either p < 0.05*, p < 0.01** or p < 0.001***.

## Results

### SVA67-lncRNA introduction into SH-SY5Y cell line

**FIGURE 2:**
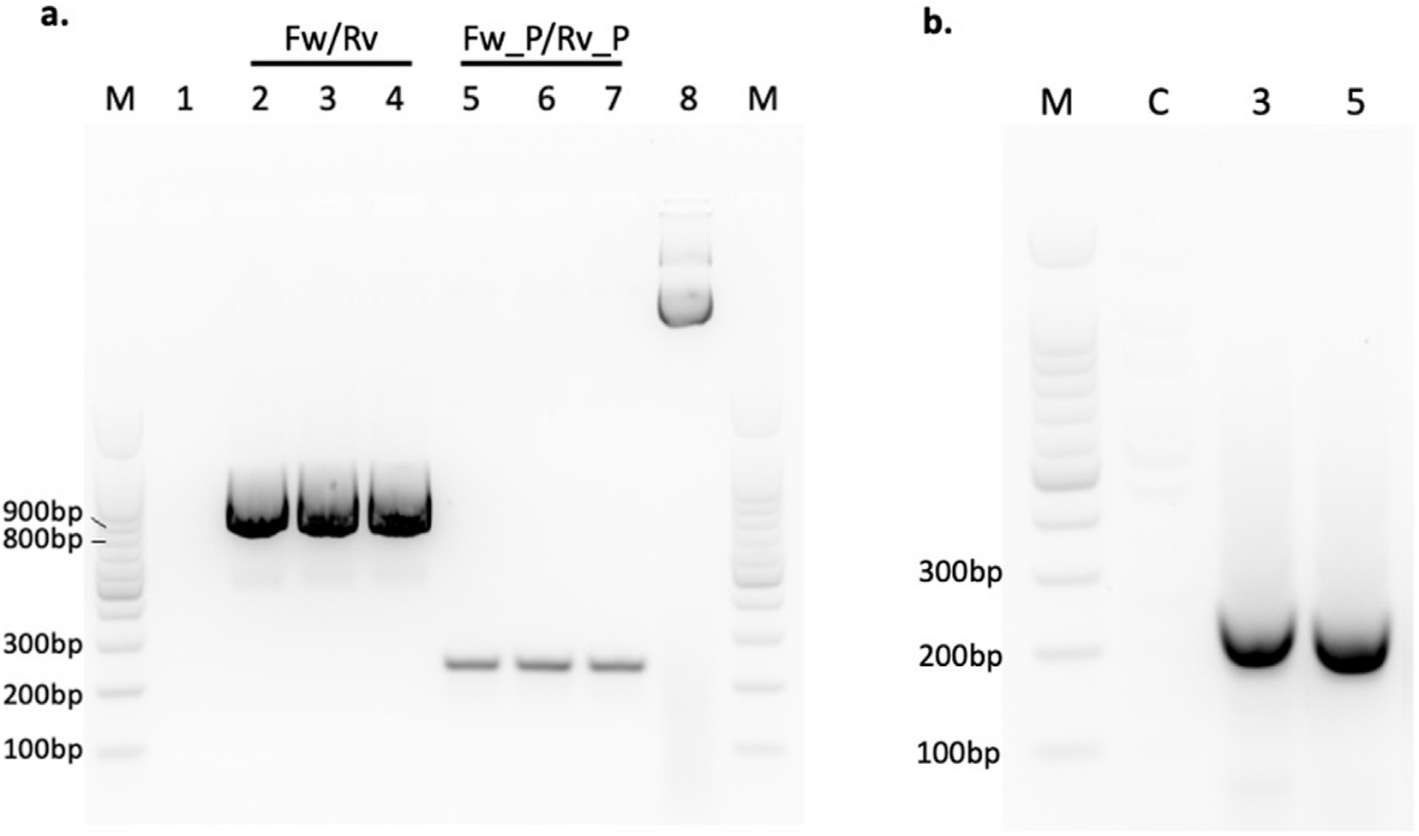
Preparation of SVA67-IncRNA and confirmation of transfection. **(a)** Image of amplicons of engineered plasmid after bacterial revival and plasmid extraction. Lane “M” represents a 100bp DNA ladder. Lane 1 represents a negative control. Fw/Rv primers expected length of 839bp was achieved as was the expected length of 210bp for Fw_P/Rv_P primers. The amplicon in lane 8 was the plasmid alone. **(b)** Image of amplicons to confirm transfection of SVA67-IncRNA into cell line. Lane “M” represents a 100bp DNA ladder. “C” represents an un-transfected sample, “3” and “5” represent the concentration of transfected SVA67-IncRNA (μg). IncRNA_SVA_67 Fw/Rv primers were used with an expected amplicon length of 241bp.

In order to assess the potential regulatory impact of SVA67-lncRNA, several target genes were identified based on presence within the SH-SY5Y cell line and proximity to the SVA_67 gene (Figure 1b). Target genes analysed included *MAPT, KANSL1, ARL17A/B, LRRC37A/2* and *NSF*. Revival of a bacterial plasmid housing the SVA67-lncRNA template was confirmed through PCR where primer pairs Fw/Rv and Fw_P/Rv_P resulted in expected band sizes of 839bp and 210bp respectively (Figure 2a). Another PCR was used to confirm the transfection of SVA67-lncRNA into SH-SY5Y cells where the results show an expected size of 241bp (Figure 2b). The expected 0bp length for control sample “C” was achieved. In total, two clonal SH-SY5Y cell populations were obtained where the SVA67-lncRNA was transfected and analysed using qPCR. Four technical replicates were used for each sample.

### SVA67-lncRNA is significantly associated with differential gene expression

**FIGURE 3:**
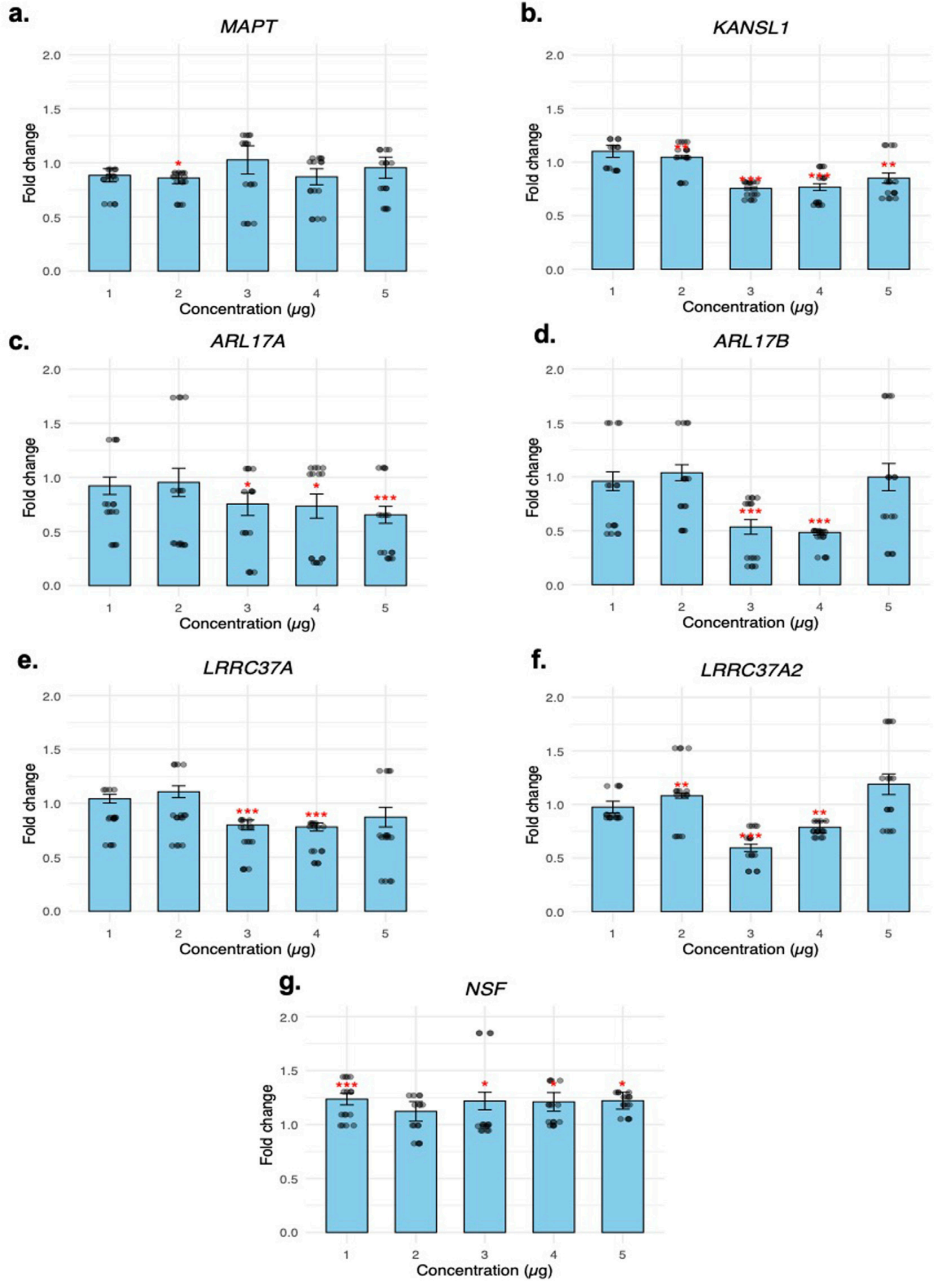
Fold change results of each target gene at increasing concentrations of transfect SVA67-IncRNA. Target gene expression results of **(a)** MAPT **(b)** KANSL1 **(c)** ARL17A **(d)** ARL17B **(e)** LRRC37A **(f)** LRRC37A2 **(g)** NSF are calculated by comparison to an empty vector sample to generate a fold change result. A Welch’s T-test was performed to determine statistical significance. *p < 0.05, **p < 0.01, ***p < 0.001. Raw fold change replicate scores are plotted using a jitter plot.

After SVA67-lncRNA was transfected and analysed, the *MAPT* gene did not show any highly significant values (Figure 3a). Analysis of the *KANSL1* gene showed significant downregulation at concentrations 3 μg and 4 µg which indicated a decrease in expression of 0.754x (p < 0.001) and 0.766x (p < 0.001) respectively (Figure 3b). Concentrations of the *ARL17A* target gene followed an inversely proportional trend where an increase in SVA67-lncRNA concentration correlated with a decrease in gene expression. The most significant fold-change value was obtained at 5 µg which resulted in a fold change of 0.654x (p < 0.001) (Figure 3c). The paralog gene of *ARL17A*, *ARL17B* showed a significant decrease in gene expression at concentrations 3 μg and 4 µg which resulted in a fold change result of 0.535x (p < 0.001) and 0.484x (p < 0.001), respectively (Figure 3d). Interestingly, *ARL17B* had the largest change in gene expression compared to the other target genes within this study with its downregulation of 0.484x. *LRRC37A* showed a similar trend to *ARL17B*, where the initial concentrations had little effect. Significant values were obtained at concentrations 3 μg and 4 µg which showed a fold change result of 0.799x (p < 0.001) and 0.781x (p < 0.001), respectively (Figure 3e). The paralog gene to *LRRC37A, LRRC37A2* showed a similar trend again to the target genes *ARL17B* and *LRRC37A* where significant downregulated gene expression was identified at 3 µg with a fold change of 0.595x (p < 0.001) (Figure 3f). *NSF* was the only target gene to be upregulated after transfection of SVA67-lncRNA. All concentration levels showed an increase in expression; however, a significant value was obtained at 1 µg which showed a fold change result of 1.23x (p < 0.001) (Figure 3g).

## Discussion

Previous studies have demonstrated associations between the *MAPT* locus H1 haplotype and PD risk [19, 20], however the specific functional variant remains underdetermined. Prior to this study, our group has shown that the presence or absence of SVA_67, located in the *MAPT* locus, correlated with the differential gene expression and progression of PD [15, 16]. This study aimed to provide preliminary evidence of transcriptional regulation caused by this SVA67-lncRNA, acknowledging the need for *in vivo* functional validation. Through the introduction of this SVA67-lncRNA at progressively increasing concentrations the expression of several genes within this target region were altered. Notable results include *NSF* being the only target gene to be upregulated and *ARL17B* having the largest change in gene expression with a fold change of 0.48x. This study provides preliminary evidence that SVA67-lncRNA has the ability to cause regulatory gene expression within the *MAPT* locus. Further studies are required regarding *in vivo* functional validation and functional assays to potentially link SVA67-lncRNA to the pathophysiological pathways found within neurodegenerative disease progression.

Both *KANSL1* and *MAPT* have been linked to neurodegenerative diseases such as Alzheimer’s disease (AD), Progressive supranuclear palsy (PSP) and PD [21–23]. *KANSL1* (KAT8 regulatory NSL complex subunit 1) is part of the NSL complex which assists KAT8 in regulating gene expression, maintaining chromatin regulation and cellular homeostasis. *KANSL1* deficiency can lead to lysosomal and autophagic dysfunction as well as increased oxidative stress, all of which are associated with PD [4]. Previous groups have shown a link between the PD risk H1 haplotype and reduced *KANSL1* mRNA expression [24–26]. It has also been shown that depletion of KAT8/KANSL1 causes significant downregulation of mitochondrial DNA transcription and translation, which eventually can lead to impaired mitochondrial respiration and oxidative stress [27]. Linda K, et al. [28] recently showed that *KANSL1* deficiency leads to impairments in lysosomal function and autophagic flux, which are associated with PD. More recently, Soutar MPM, et al. [24] hypothesised that less severe changes in KAT8/KANSL1 and its associated mitochondrial defects may lead to accumulation of cellular damage. This can result in selective vulnerability of dopaminergic neurons at a later stage in life. Past studies have linked the decrease in gene expression of *KANSL1* to many dysfunctional pathways that are associated with PD [26, 29]. We identified in this study that *KANSL1* gene expression is downregulated at specific concentrations of SVA67-lncRNA. These findings could potentially associate this SVA67-lncRNA and PD progression, requiring the need for functional assays and validation.

Tau is a microtubule-associated protein encoded by the *MAPT* gene that has important functions in binding and stabilising the cytoskeleton, regulating cellular transport and interacting with other cellular structures such as cytoplasmic organelles, plasma membrane and the nucleus [30]. Tau is the most commonly deposited protein in the aging brain and in neurodegenerative diseases, including PD [29]. Aggregation of tau is a hallmark of many neurodegenerative diseases (e.g., PD), however there remains the need for clarification and further research to associate the *MAPT* gene to PD. The results of this study show a slight decrease in expression over several different concentrations when introduced with the SVA67-lncRNA. This highlights a small regulatory effect in relation to the *MAPT* gene and this complex, however it is difficult to associate this with PD progression due to the low gene expression fold-change and limited current research associating altered *MAPT* gene expression to PD.

*ARL17A* and its paralog *ARL17B* are genes that belong to the sub-family of ADP-ribosylation factor-like (ARF-like) genes, which are part of the ARF family that regulates membrane trafficking and vesicular transport [30]. Tian et al. [31] found that *ARL17B* was associated with negative control of neuron projection development. Alvarado CX et al. [32] identified several genes to be individually significant in multiple different omics for AD, PD and PSP. These included *MAPT, CRHR1, KANSL1* and *ARL17A*. Finally, a study performed by Jiayang Li et al. [33] using RNA-seq showed that downregulated gene expression in *ARL17A, ARL17B* and *NSF* was correlated with PD risk. Results from the current study showed a decrease in gene expression for *ARL17A* and *ARL17B.* The former showed several significant decreased fold-change scores, and the latter showed a significant fold-change decrease at concentrations 3 µg (0.54x) and 4 µg (0.48x). Limited work has been accomplished to fully understand the function of the genes *ARL17A/B* and linking them to disease progression. Our study found that the expression levels of both genes were downregulated at specific concentrations of the transfected SVA67-lncRNA. However, due to the limited research linking *ARL17A/B* gene expression to PD, it is difficult to directly associate these findings with PD progression. Nevertheless, these results contribute to the growing body of evidence suggesting a potential connection between *ARL17A/B* and the progression of PD.

*LRRC37A* and *LRRC37A2* encode the proteins Leucine-rich repeat-containing protein 37A and 37A2, respectively. These genes are paralogs of each other and have complex structural variation resulting in difficulty when analysing gene function and association to disease phenotypes. Although little is known about the function of *LRRC37A/2*, they have been associated with cellular migration and synapse formation [34], and immune and inflammatory response [35]. Shani S, et al. [36] performed whole-genome sequencing on unrelated individuals with PD from an Ashkenazi Jewish population, using brain tissue samples, they performed eQTL analysis and identifying a link between the H2 haplotype and increased *LRRC37A/2* RNA expression, pointing to a possible role in PD pathology. Their findings also indicated that *LRRC37A2* shows higher expression in both neurons and glial cells compared to *LRRC37A*, suggesting it may play a more prominent role in disease progression. Conversely, research by Bowles et al. [37] reported that protective H1 sub-haplotypes were associated with elevated *LRRC37A/2* expression. Although both studies suggest a relationship between *LRRC37A/2* and PD, they associate increased expression with different haplotypes. Our study identified a decrease in gene expression following the introduction of the SVA67-lncRNA at certain concentrations. It is difficult to correlate this finding with an association with PD as current literature is conflicting and minimal. Nevertheless, it was demonstrated that the SVA67-lncRNA effected gene expression of the gene paralogs *LRRC37A* and *LRRC37A2*.

The *NSF* gene encodes the protein N-ethylmaleimide-sensitive factor, a gene associated with ATPase activity essential for driving the disassembly of soluble NSF-attachment protein receptor (SNARE) complexes. This activity is critical for intracellular vesicle transport and intercellular substance transfer. In addition to its role in vesicle trafficking, *NSF* has been shown to interact with dopamine receptors [38]. Dysfunctional *NSF* activity has been linked to several neurological disorders, including PD [39], AD [40], and epilepsy [39]. For instance, reduced *NSF* levels can hinder autophagy, a key process responsible for the removal of cellular waste and the maintenance of cellular homeostasis. Disrupted autophagy can lead to the build-up of pathological proteins, a hallmark of diseases such as PD and AD. Interestingly, patients in early stages of PD have reported elevated *NSF* gene expression [41]. Although numerous studies have associated *NSF* with neurological disorders like PD and AD, there is still a shortage of research specifically focusing on variable gene expression. In our study, we observed an increase in gene expression after the SVA67-lncRNA was transfected. However, given the limited existing literature on *NSF* gene expression effects, it is difficult to correlate this result to a disease phenotype. It is interesting to note that this target gene was the only target gene to be upregulated in response to the SVA67-lncRNA transfection.

The gene-specific differences observed among the target genes of this study suggest that the regulatory influence of SVA67-lncRNA depends on local genomic and epigenetic context. This is consistent with previous studies showing that retrotransposon derived lncRNAs can modulate gene expression through chromatin remodelling and locus-specific interactions [13, 14, 16, 42]. While the trends reported here were consistent across biological replicates, the limited sample size does not rule out subtle experimental variation. Future studies incorporating additional replicates and protein-level validation are needed to confirm these findings. Notably, the *MAPT* locus is known to exhibit complex epigenetic regulation, including allele-specific methylation and histone modifications that differ between the H1 and H2 haplotypes [19, 20, 43]. These epigenetic features may contribute to the concentration-dependent transcriptional effects observed following the introduction of SVA67-lncRNA. Collectively, these findings indicate that SVA67-lncRNA influences gene expression within the *MAPT* locus, although further studies are required to determine whether SVA67-lncRNA acts as a cis-regulatory or trans-regulatory element and whether effects are locus-specific and whether they involve epigenetic or chromatin-associated mechanisms.

## Conclusion

This study investigated the functional impact of SVA67-lncRNA within the *MAPT* locus, demonstrating that its introduction alters the expression of target genes, as initially hypothesized. These findings lay the groundwork for future research into the potential role of SVA67-lncRNA in neurological disease progression. Given its broad regulatory influence and limited biological replicates, further investigation is warranted to explore whether this complex exerts similar effects at other loci across the human genome. Correlating results from this study to previous GWAS studies highlight a potential link between this SVA67-lncRNA and PD progression. However, further functional assays and *in-vivo* validation is required. Further studies could involve a western blot analysis to provide protein-level validation of the transcriptional changes observed in this study. Functional studies should be performed to understand the effect this SVA67-lncRNA has on a phenotypic level. For example, the target gene ARL17B showed a significant down regulation in this study. It would be beneficial to measure if the cells GTPase activity is also affected as this is the main function of this gene and is a contributing mechanism to PD progression. Nevertheless, this study supports the notion that this SVA67-lncRNA has regulatory effects on several genes within the *MAPT* locus. Moreover, it underscores the broader significance of TEs and lncRNAs in addressing the missing heritability of neurodegenerative diseases. Overall, this research emphasizes the critical need to further explore non-coding genomic elements and their roles in the aetiology of neurodegenerative disorders.

## Data Availability

The original contributions presented in the study are included in the article/Supplementary Material, further inquiries can be directed to the corresponding author.

## References

[1] Dorsey ER Bloem BR . The parkinson Pandemic-A call to action. JAMA Neurol (2018) 75(1):9–10. 10.1001/jamaneurol.2017.3299 29131880

[2] GBD 2016 Parkinson's Disease Collaborators. Global, regional, and national burden of parkinson's disease, 1990-2016: a systematic analysis for the global burden of disease study 2016. Lancet Neurol (2021) 20(12):e7. 10.1016/S1474-4422(21)00382-3

[3] Emamzadeh FN Surguchov A . Parkinson's disease: biomarkers, treatment, and risk factors. Front Neurosci (2018) 12:612. 10.3389/fnins.2018.00612 30214392 PMC6125353

[4] Drobny A Boros FA Balta D Prieto Huarcaya S Caylioglu D Qazi N Reciprocal effects of alpha-synuclein aggregation and lysosomal homeostasis in synucleinopathy models. Transl Neurodegener (2023) 12(1):31. 10.1186/s40035-023-00363-z 37312133 PMC10262594

[5] Ganjam GK Bolte K Matschke LA Neitemeier S Dolga AM Höllerhage M Mitochondrial damage by α-synuclein causes cell death in human dopaminergic neurons. Cell Death Dis (2019) 10:865. 10.1038/s41419-019-2091-2 31727879 PMC6856124

[6] Ebanks K Lewis PA Bandopadhyay R . Vesicular dysfunction and the pathogenesis of parkinson's disease: clues from genetic studies. Front Neurosci (2020) 13:1381. 10.3389/fnins.2019.01381 31969802 PMC6960401

[7] Tran J Anastacio H Bardy C . Genetic predispositions of Parkinson’s disease revealed in patient-derived brain cells. Npj Parkinsons Dis (2020) 6:8. 10.1038/s41531-020-0110-8 32352027 PMC7181694

[8] Buniello A MacArthur JAL Cerezo M Harris LW Hayhurst J Malangone C The NHGRI-EBI GWAS catalog of published genome-wide association studies, targeted arrays and summary statistics 2019. Nucleic Acids Res (2019) 47(D1):D1005–D1012. 10.1093/nar/gky1120 30445434 PMC6323933

[9] Aleknonytė-Resch M Trinh J Leonard H Delcambre S Leitão E Lai D Genome-wide case-only analysis of gene-gene interactions with known parkinson's disease risk variants reveals link between LRRK2 and SYT10. NPJ Parkinsons Dis (2023) 9(1):102. 10.1038/s41531-023-00550-9 37386035 PMC10310744

[10] Yu WJ Cheng L Li NN Wang L Tan EK Peng R . Interaction between SNCA, LRRK2 and GAK increases susceptibility to parkinson's disease in a Chinese population. eNeurologicalSci (2015) 1(1):3–6. 10.1016/j.ensci.2015.08.001 29479569 PMC5852682

[11] Nalls MA Blauwendraat C Vallerga CL Heilbron K Bandres-Ciga S Chang D Identification of novel risk loci, causal insights, and heritable risk for parkinson's disease: a meta-analysis of genome-wide association studies. The Lancet Neurol (2019) 18(12):1091–102. 10.1016/S1474-4422(19)30320-5 31701892 PMC8422160

[12] Kim JJ Vitale D Otani DV Lian MM Heilbron K Aslibekyan S Multi-ancestry genome-wide association meta-analysis of parkinson's disease. Nat Genet (2024) 56(1):27–36. 10.1038/s41588-023-01584-8 38155330 PMC10786718

[13] Lawson HA Liang Y Wang T . Transposable elements in Mammalian chromatin organization. Nat Rev Genet (2023) 24:712–23. 10.1038/s41576-023-00609-6 37286742

[14] Gianfrancesco O Geary B Savage AL Billingsley KJ Bubb VJ Quinn JP . The role of SINE-VNTR-Alu (SVA) retrotransposons in shaping the human genome. Int J Mol Sci (2019) 20(23):5977. 10.3390/ijms20235977 31783611 PMC6928650

[15] Pfaff AL Bubb VJ Quinn JP Koks S . Reference SVA insertion polymorphisms are associated with parkinson's disease progression and differential gene expression. NPJ Parkinsons Dis (2021) 7(1):44. 10.1038/s41531-021-00189-4 34035310 PMC8149882

[16] Fröhlich A Hughes LS Middlehurst B Pfaff AL Bubb VJ Koks S CRISPR deletion of a SINE-VNTR-Alu (SVA_67) retrotransposon demonstrates its ability to differentially modulate gene expression at the MAPT locus. Front Neurol (2023) 14:1273036. 10.3389/fneur.2023.1273036 37840928 PMC10570551

[17] Frohlich A Pfaff AL Bubb VJ Koks S Quinn JP . Characterisation of the function of a SINE-VNTR-Alu retrotransposon to modulate isoform expression at the MAPT locus. Front Mol Neurosci (2022) 15:815695. 10.3389/fnmol.2022.815695 35370538 PMC8965460

[18] Wider C Vilariño-Güell C Jasinska-Myga B Heckman MG Soto-Ortolaza AI Cobb SA Association of the MAPT locus with parkinson's disease. Eur J Neurol (2010) 17(3):483–6. 10.1111/j.1468-1331.2009.02847.x 19912324 PMC3906622

[19] Desikan RS Schork AJ Wang Y Witoelar A Sharma M McEvoy LK Genetic overlap between Alzheimer’s disease and Parkinson’s disease at the MAPT locus. Mol Psychiatry (2015) 20(12):1588–95. 10.1038/mp.2015.6 25687773 PMC4539304

[20] Bowles KR Pugh DA Liu Y Patel T Renton AE Bandres-Ciga S 17q21.31 sub-haplotypes underlying H1-associated risk for parkinson's disease are associated with LRRC37A/2 expression in astrocytes. Mol Neurodegeneration (2022) 17(1):48. 10.1186/s13024-022-00551-x 35841044 PMC9284779

[21] Müller U Höglinger G Dickson DW . Multifactorial etiology of progressive supranuclear palsy (PSP): the genetic component. Acta Neuropathol (2025) 149(1):58. 10.1007/s00401-025-02898-z 40465013 PMC12137433

[22] Wainberg M Andrews SJ Tripathy SJ . Shared genetic risk loci between alzheimer's disease and related dementias, parkinson's disease, and amyotrophic lateral sclerosis. Alzheimer's Res & Ther (2023) 15(1):113. 10.1186/s13195-023-01244-3 37328865 PMC10273745

[23] NCBI. KANSL1 KAT8 regulatory NSL complex subunit 1 (*Homo sapiens)* gene ID: 284058. (2025).

[24] Ferrari R Wang Y Vandrovcova J Guelfi S Witeolar A Karch CM Genetic architecture of sporadic frontotemporal dementia and overlap with Alzheimer’s and Parkinson’s diseases. J Neurol Neurosurg Psychiatry (2017) 88:152–64. 10.1136/jnnp-2016-314411 27899424 PMC5237405

[25] Koks S Pfaff AL Bubb VJ Quinn JP . Transcript variants of genes involved in neurodegeneration are differentially regulated by the APOE and MAPT haplotypes. Genes (2021) 12:423. 10.3390/genes12030423 33804213 PMC7999745

[26] Soutar MPM Melandri D O’Callaghan B Annuario E Monaghan AE Welsh NJ Regulation of mitophagy by the NSL complex underlies genetic risk for parkinson's disease at 16q11.2 and MAPT H1 loci. Brain (2022) 145(12):4349–67. 10.1093/brain/awac325 36074904 PMC9762952

[27] Chatterjee A Seyfferth J Lucci J Gilsbach R Preissl S Böttinger L MOF acetyl transferase regulates transcription and respiration in mitochondria. Cell (2016) 167:722–38.e23. 10.1016/j.cell.2016.09.052 27768893

[28] Linda K Lewerissa EI Verboven AHA Gabriele M Frega M Klein Gunnewiek TM Imbalanced autophagy causes synaptic deficits in a human model for neurodevelopmental disorders. Autophagy (2021) 18(2):423–42. 10.1080/15548627.2021.1936777 34286667 PMC8942553

[29] Hicks AR Reynolds RH O’Callaghan B García-Ruiz S Gil-Martínez AL Botía J The non-specific lethal complex regulates genes and pathways genetically linked to parkinson's disease. Brain (2023) 146(12):4974–87. 10.1093/brain/awad246 37522749 PMC10689904

[30] Pan L Meng L He M Zhang Z . Tau in the pathophysiology of parkinson's disease. J Mol Neurosci (2021) 71(11):2179–91. 10.1007/s12031-020-01776-5 33459970 PMC8585831

[31] Tian Y Ma G Li H Zeng Y Zhou S Wang X Shared genetics and comorbid genes of amyotrophic lateral sclerosis and Parkinson’s disease. Movement Disord (2023) 38:1813–21. 10.1002/mds.29572 37534731

[32] Alvarado CX Makarious MB Weller CA Vitale D Koretsky MJ Bandres-Ciga S omicSynth: an open multi-omic community resource for identifying druggable targets across neurodegenerative diseases. The Am J Hum Genet (2024) 111(1):150–64. 10.1016/j.ajhg.2023.12.006 38181731 PMC10806756

[33] Li J Amoh BK McCormick E Tarkunde A Zhu KF Perez A Integration of transcriptome-wide association study with neuronal dysfunction assays provides functional genomics evidence for parkinson's disease genes. Hum Mol Genet (2023) 32(4):685–95. 10.1093/hmg/ddac230 36173927 PMC9896475

[34] Giannuzzi G Siswara P Malig M Marques-Bonet T Mullikin JC Ventura M Evolutionary dynamism of the primate LRRC37 gene family. Genome Res (2013) 23:46–59. 10.1101/gr.138842.112 23064749 PMC3530683

[35] Falola MI Wiener HW Wineinger NE Cutter GR Kimberly RP Edberg JC Genomic copy number variants: evidence for association with antibody response to anthrax vaccine adsorbed. PLoS ONE (2013) 8:e64813. 10.1371/journal.pone.0064813 23741398 PMC3669407

[36] Shani S Gana-Weisz M Bar-Shira A Thaler A Gurevich T Mirelman A *MAPT* locus in parkinson's disease patients of Ashkenazi origin: a stratified analysis. Genes (Basel) (2023) 15(1):46. 10.3390/genes15010046 38254936 PMC10815687

[37] Bao H Das D Courtney NA Jiang Y Briguglio JS Lou X Dynamics and number of trans-SNARE complexes determine nascent fusion pore properties. Nature (2018) 554:260–3. 10.1038/nature25481 29420480 PMC5808578

[38] Belluzzi E Gonnelli A Cirnaru MD Marte A Plotegher N Russo I LRRK2 phosphorylates pre-synaptic N-ethylmaleimide sensitive fusion (NSF) protein enhancing its ATPase activity and SNARE complex disassembling rate. Mol Neurodegeneration (2016) 11:1. 10.1186/s13024-015-0066-z 26758690 PMC4711005

[39] Herold C Bidmon HJ Pannek HW Hans V Gorji A Speckmann EJ ATPase N-ethylmaleimide-sensitive fusion protein: a novel key player for causing spontaneous network excitation in human temporal lobe epilepsy. Neuroscience (2018) 371:371–83. 10.1016/j.neuroscience.2017.12.013 29262299

[40] Hashimoto S Matsuba Y Kamano N Mihira N Sahara N Takano J Tau binding protein CAPON induces tau aggregation and neurodegeneration. Nat Commun (2019) 10:2394. 10.1038/s41467-019-10278-x 31160584 PMC6546774

[41] Semenova EI Partevian SA Shulskaya MV Rudenok MM Lukashevich MV Baranova NM Analysis of *ADORA2A, MTA1, PTGDS, PTGS2, NSF*, and *HNMT* gene expression levels in peripheral blood of patients with early stages of parkinson's disease. Biomed Res Int (2023) 2023:9412776. 10.1155/2023/9412776 38027039 PMC10681775

[42] Nadler MJS Chang W Ozkaynak E Huo Y Nong Y Boillot M Hominoid SVA-lncRNA AK057321 targets human-specific SVA retrotransposons in SCN8A and CDK5RAP2 to initiate neuronal maturation. Commun Biol (2023) 6(1):347. 10.1038/s42003-023-04683-8 36997626 PMC10063665

[43] Zody MC Jiang Z Fung HC Antonacci F Hillier LW Cardone MF Evolutionary toggling of the MAPT 17q21.31 inversion region. Nat Genet (2008) 40(9):1076–83. 10.1038/ng.193 19165922 PMC2684794

