# Supplementary material for: Characterisation of the function of a lncRNA containing SINE-VNTR-Alu 67 to regulate the genes at the MAPT locus
Source: Exp Biol Med (Maywood). 2025 Nov 27;250:10805. doi: 10.3389/ebm.2025.10805 (PMC12695722; doi:10.3389/ebm.2025.10805)
Supplement: Supplementary file 1 [file Table1.docx]

**Supplementary material**

Table 1. Primer sequences and cycling conditions for PCR analysis (synthesised by IDT Pty Ltd.)


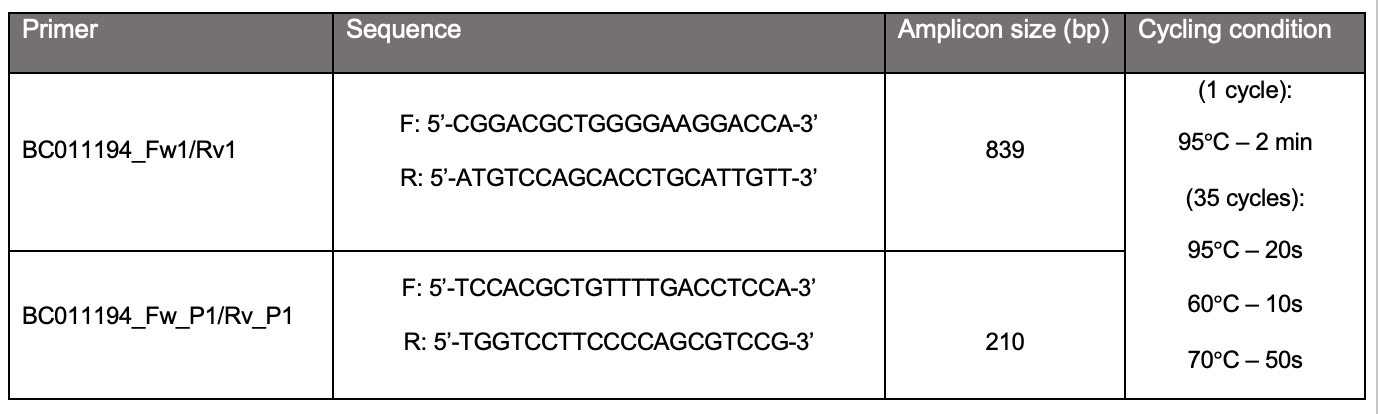


Table 2. Primer sequences and cycling conditions for qPCR analysis (synthesised by IDT Pty Ltd.)


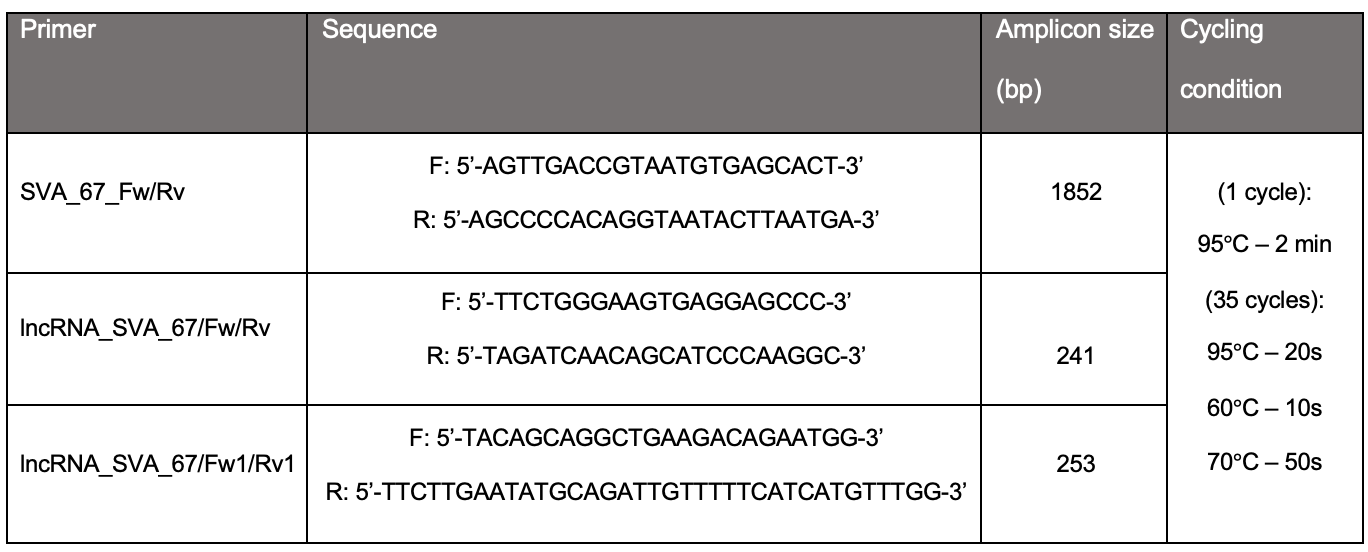


| **Target Gene**  Table 3. qPCR analysis of target gene expression. | **Experiment** | **Concentration (µg)** | **E_Control** | **Repeat** | **Ct_Target_Gene** | **Ct_Control** |
| --- | --- | --- | --- | --- | --- | --- |
| **MAPT** | 1 | 1 | G | 1 | 32.53 | 26.08 |
| **MAPT** | 1 | 1 | G | 2 | 32.64 | 26.08 |
| **MAPT** | 1 | 1 | G | 3 | 32.61 | 26.03 |
| **MAPT** | 1 | 1 | G | 4 | 32.8 | 26 |
| **MAPT** | 1 | 1 | H | 1 | 33.3 | 30.26 |
| **MAPT** | 1 | 1 | H | 2 | 33.78 | 30.27 |
| **MAPT** | 1 | 1 | H | 3 | 33.29 | 30.17 |
| **MAPT** | 1 | 1 | H | 4 | 33.28 | 29.93 |
| **MAPT** | 1 | 2 | G | 1 | 33.13 | 26.36 |
| **MAPT** | 1 | 2 | G | 2 | 32.62 | 26.25 |
| **MAPT** | 1 | 2 | G | 3 | 32.88 | 26.19 |
| **MAPT** | 1 | 2 | G | 4 | 32.89 | 26.31 |
| **MAPT** | 1 | 2 | H | 1 | 33.52 | 30.32 |
| **MAPT** | 1 | 2 | H | 2 | 33.42 | 30.33 |
| **MAPT** | 1 | 2 | H | 3 | 33.71 | 30.41 |
| **MAPT** | 1 | 2 | H | 4 | 33.75 | 30.51 |
| **MAPT** | 1 | 3 | G | 1 | 33.28 | 26.67 |
| **MAPT** | 1 | 3 | G | 2 | 33.59 | 26.39 |
| **MAPT** | 1 | 3 | G | 3 | 33.25 | 26.24 |
| **MAPT** | 1 | 3 | G | 4 | 33.83 | 26.28 |
| **MAPT** | 1 | 3 | H | 1 | 33.98 | 30.77 |
| **MAPT** | 1 | 3 | H | 2 | 33.92 | 30.48 |
| **MAPT** | 1 | 3 | H | 3 | 33.98 | 30.53 |
| **MAPT** | 1 | 3 | H | 4 | 33.88 | 30.42 |
| **MAPT** | 1 | 4 | G | 1 | 33.89 | 26.84 |
| **MAPT** | 1 | 4 | G | 2 | 33.82 | 26.79 |
| **MAPT** | 1 | 4 | G | 3 | 33.51 | 26.52 |
| **MAPT** | 1 | 4 | G | 4 | 33.31 | 26.5 |
| **MAPT** | 1 | 4 | H | 1 | 33.91 | 31.05 |
| **MAPT** | 1 | 4 | H | 2 | 34.42 | 31.15 |
| **MAPT** | 1 | 4 | H | 3 | 34.05 | 30.88 |
| **MAPT** | 1 | 4 | H | 4 | 33.79 | 31.05 |
| **MAPT** | 1 | 5 | G | 1 | 33.49 | 26.53 |
| **MAPT** | 1 | 5 | G | 2 | 32.96 | 26.4 |
| **MAPT** | 1 | 5 | G | 3 | 33.07 | 26.53 |
| **MAPT** | 1 | 5 | G | 4 | 33.34 | 26.61 |
| **MAPT** | 1 | 5 | H | 1 | 34.1 | 30.72 |
| **MAPT** | 1 | 5 | H | 2 | 33.96 | 30.52 |
| **MAPT** | 1 | 5 | H | 3 | 34.19 | 30.8 |
| **MAPT** | 1 | 5 | H | 4 | 33.97 | 30.35 |
| **KANSL1** | 1 | 1 | G | 1 | 29.18 | 26.15 |
| **KANSL1** | 1 | 1 | G | 2 | 29.19 | 26.11 |
| **KANSL1** | 1 | 1 | G | 3 | 29.1 | 26.1 |
| **KANSL1** | 1 | 1 | G | 4 | 28.95 | 25.9 |
| **KANSL1** | 1 | 1 | H | 1 | 30.23 | 30.15 |
| **KANSL1** | 1 | 1 | H | 2 | 30.42 | 30.17 |
| **KANSL1** | 1 | 1 | H | 3 | 30.2 | 29.93 |
| **KANSL1** | 1 | 1 | H | 4 | 30.11 | 30.04 |
| **KANSL1** | 1 | 2 | G | 1 | 29.57 | 26.42 |
| **KANSL1** | 1 | 2 | G | 2 | 29.51 | 26.33 |
| **KANSL1** | 1 | 2 | G | 3 | 29.52 | 26.36 |
| **KANSL1** | 1 | 2 | G | 4 | 29.55 | 26.37 |
| **KANSL1** | 1 | 2 | H | 1 | 30.48 | 30.45 |
| **KANSL1** | 1 | 2 | H | 2 | 29.91 | 30 |
| **KANSL1** | 1 | 2 | H | 3 | 30.33 | 30.41 |
| **KANSL1** | 1 | 2 | H | 4 | 30.38 | 30.28 |
| **KANSL1** | 1 | 3 | G | 1 | 29.64 | 25.94 |
| **KANSL1** | 1 | 3 | G | 2 | 29.54 | 25.91 |
| **KANSL1** | 1 | 3 | G | 3 | 29.43 | 25.81 |
| **KANSL1** | 1 | 3 | G | 4 | 29.44 | 25.88 |
| **KANSL1** | 1 | 3 | H | 1 | 30.89 | 30.59 |
| **KANSL1** | 1 | 3 | H | 2 | 30.73 | 30.52 |
| **KANSL1** | 1 | 3 | H | 3 | 30.91 | 30.52 |
| **KANSL1** | 1 | 3 | H | 4 | 30.95 | 30.47 |
| **KANSL1** | 1 | 4 | G | 1 | 29.84 | 26.23 |
| **KANSL1** | 1 | 4 | G | 2 | 29.85 | 26.36 |
| **KANSL1** | 1 | 4 | G | 3 | 29.93 | 26.47 |
| **KANSL1** | 1 | 4 | G | 4 | 29.93 | 26.28 |
| **KANSL1** | 1 | 4 | H | 1 | 31.09 | 31.11 |
| **KANSL1** | 1 | 4 | H | 2 | 31.11 | 30.92 |
| **KANSL1** | 1 | 4 | H | 3 | 31.06 | 30.91 |
| **KANSL1** | 1 | 4 | H | 4 | 31.1 | 30.99 |
| **KANSL1** | 1 | 5 | G | 1 | 30.01 | 27.06 |
| **KANSL1** | 1 | 5 | G | 2 | 29.97 | 26.77 |
| **KANSL1** | 1 | 5 | G | 3 | 29.64 | 26.52 |
| **KANSL1** | 1 | 5 | G | 4 | 29.69 | 26.52 |
| **KANSL1** | 1 | 5 | H | 1 | 31.04 | 30.72 |
| **KANSL1** | 1 | 5 | H | 2 | 30.83 | 30.39 |
| **KANSL1** | 1 | 5 | H | 3 | 30.79 | 30.54 |
| **KANSL1** | 1 | 5 | H | 4 | 30.82 | 30.47 |
| **ARL17A** | 1 | 1 | G | 1 | 34.21 | 25.99 |
| **ARL17A** | 1 | 1 | G | 2 | 34.7 | 25.87 |
| **ARL17A** | 1 | 1 | G | 3 | 33.75 | 25.77 |
| **ARL17A** | 1 | 1 | G | 4 | 34.18 | 25.81 |
| **ARL17A** | 1 | 1 | H | 1 | 34.73 | 29.31 |
| **ARL17A** | 1 | 1 | H | 2 | 33.92 | 29.32 |
| **ARL17A** | 1 | 1 | H | 3 | 34.46 | 29.57 |
| **ARL17A** | 1 | 1 | H | 4 | 34.42 | 29.39 |
| **ARL17A** | 1 | 2 | G | 1 | 34.12 | 26.1 |
| **ARL17A** | 1 | 2 | G | 2 | 34.25 | 26.09 |
| **ARL17A** | 1 | 2 | G | 3 | 34.29 | 26.1 |
| **ARL17A** | 1 | 2 | G | 4 | 33.61 | 26.12 |
| **ARL17A** | 1 | 2 | H | 1 | 33.63 | 29.42 |
| **ARL17A** | 1 | 2 | H | 2 | 34.64 | 29.73 |
| **ARL17A** | 1 | 2 | H | 3 | 34.72 | 29.42 |
| **ARL17A** | 1 | 2 | H | 4 | 34.11 | 29.47 |
| **ARL17A** | 1 | 3 | G | 1 | 34.56 | 25.87 |
| **ARL17A** | 1 | 3 | G | 2 | 35.39 | 25.93 |
| **ARL17A** | 1 | 3 | G | 3 | 34.65 | 25.93 |
| **ARL17A** | 1 | 3 | G | 4 | 34.99 | 26.1 |
| **ARL17A** | 1 | 3 | H | 1 | 34.37 | 29.94 |
| **ARL17A** | 1 | 3 | H | 2 | 34.11 | 29.72 |
| **ARL17A** | 1 | 3 | H | 3 | 34.2 | 30.06 |
| **ARL17A** | 1 | 3 | H | 4 | 34.65 | 29.75 |
| **ARL17A** | 1 | 4 | G | 1 | 34.72 | 26.17 |
| **ARL17A** | 1 | 4 | G | 2 | 34.76 | 26.18 |
| **ARL17A** | 1 | 4 | G | 3 | 34.69 | 26.07 |
| **ARL17A** | 1 | 4 | G | 4 | 34.9 | 26.07 |
| **ARL17A** | 1 | 4 | H | 1 | 34.46 | 30.36 |
| **ARL17A** | 1 | 4 | H | 2 | 34.79 | 30.38 |
| **ARL17A** | 1 | 4 | H | 3 | 35.21 | 30.49 |
| **ARL17A** | 1 | 4 | H | 4 | 35.21 | 30.32 |
| **ARL17A** | 1 | 5 | G | 1 | 34.92 | 26.61 |
| **ARL17A** | 1 | 5 | G | 2 | 35.17 | 26.46 |
| **ARL17A** | 1 | 5 | G | 3 | 35 | 26.24 |
| **ARL17A** | 1 | 5 | G | 4 | 35.14 | 26.38 |
| **ARL17A** | 1 | 5 | H | 1 | 34.5 | 29.83 |
| **ARL17A** | 1 | 5 | H | 2 | 35.51 | 29.63 |
| **ARL17A** | 1 | 5 | H | 3 | 34.4 | 29.9 |
| **ARL17A** | 1 | 5 | H | 4 | 35.22 | 29.52 |
| **ARL17B** | 1 | 1 | G | 1 | 34.03 | 23.21 |
| **ARL17B** | 1 | 1 | G | 2 | 34.15 | 23.04 |
| **ARL17B** | 1 | 1 | G | 3 | 33.66 | 23.07 |
| **ARL17B** | 1 | 1 | G | 4 | 33.65 | 23.1 |
| **ARL17B** | 1 | 1 | H | 1 | 34.54 | 29.2 |
| **ARL17B** | 1 | 1 | H | 2 | 34.13 | 29.18 |
| **ARL17B** | 1 | 1 | H | 3 | 34.01 | 29.04 |
| **ARL17B** | 1 | 1 | H | 4 | 34.14 | 29.21 |
| **ARL17B** | 1 | 2 | G | 1 | 34.03 | 23.28 |
| **ARL17B** | 1 | 2 | G | 2 | 34.11 | 23.45 |
| **ARL17B** | 1 | 2 | G | 3 | 33.94 | 23.37 |
| **ARL17B** | 1 | 2 | G | 4 | 34.28 | 23.3 |
| **ARL17B** | 1 | 2 | H | 1 | 34.57 | 29.44 |
| **ARL17B** | 1 | 2 | H | 2 | 34.07 | 29.47 |
| **ARL17B** | 1 | 2 | H | 3 | 34.41 | 29.35 |
| **ARL17B** | 1 | 2 | H | 4 | 34.35 | 29.31 |
| **ARL17B** | 1 | 3 | G | 1 | 35.17 | 23.08 |
| **ARL17B** | 1 | 3 | G | 2 | 34.79 | 23.12 |
| **ARL17B** | 1 | 3 | G | 3 | 35.31 | 23.08 |
| **ARL17B** | 1 | 3 | G | 4 | 34.55 | 23.09 |
| **ARL17B** | 1 | 3 | H | 1 | 34.79 | 29.48 |
| **ARL17B** | 1 | 3 | H | 2 | 34.99 | 29.57 |
| **ARL17B** | 1 | 3 | H | 3 | 34.62 | 29.64 |
| **ARL17B** | 1 | 3 | H | 4 | 35.06 | 29.79 |
| **ARL17B** | 1 | 4 | G | 1 | 35.93 | 23.06 |
| **ARL17B** | 1 | 4 | G | 2 | 35.3 | 23 |
| **ARL17B** | 1 | 4 | G | 3 | 35.38 | 23.1 |
| **ARL17B** | 1 | 4 | G | 4 | 35.98 | 22.98 |
| **ARL17B** | 1 | 4 | H | 1 | 36.42 | 30.36 |
| **ARL17B** | 1 | 4 | H | 2 | 36.68 | 30.4 |
| **ARL17B** | 1 | 4 | H | 3 | 36.19 | 30.33 |
| **ARL17B** | 1 | 4 | H | 4 | 36.44 | 30.26 |
| **ARL17B** | 1 | 5 | G | 1 | 34.08 | 24.12 |
| **ARL17B** | 1 | 5 | G | 2 | 34.56 | 23.83 |
| **ARL17B** | 1 | 5 | G | 3 | 34.56 | 23.83 |
| **ARL17B** | 1 | 5 | G | 4 | 34.29 | 23.58 |
| **ARL17B** | 1 | 5 | H | 1 | 34.22 | 29.69 |
| **ARL17B** | 1 | 5 | H | 2 | 34.39 | 29.58 |
| **ARL17B** | 1 | 5 | H | 3 | 34.81 | 29.46 |
| **ARL17B** | 1 | 5 | H | 4 | 34.63 | 29.59 |
| **LRRC37A** | 1 | 1 | G | 1 | 32.71 | 25.74 |
| **LRRC37A** | 1 | 1 | G | 2 | 32.8 | 25.65 |
| **LRRC37A** | 1 | 1 | G | 3 | 32.71 | 25.64 |
| **LRRC37A** | 1 | 1 | G | 4 | 32.81 | 25.56 |
| **LRRC37A** | 1 | 1 | H | 1 | 31.75 | 29.15 |
| **LRRC37A** | 1 | 1 | H | 2 | 31.73 | 29.18 |
| **LRRC37A** | 1 | 1 | H | 3 | 32.06 | 29.19 |
| **LRRC37A** | 1 | 1 | H | 4 | 31.88 | 29.13 |
| **LRRC37A** | 1 | 2 | G | 1 | 32.45 | 25.88 |
| **LRRC37A** | 1 | 2 | G | 2 | 32.69 | 25.69 |
| **LRRC37A** | 1 | 2 | G | 3 | 32.82 | 25.72 |
| **LRRC37A** | 1 | 2 | G | 4 | 32.48 | 25.74 |
| **LRRC37A** | 1 | 2 | H | 1 | 32.38 | 29.53 |
| **LRRC37A** | 1 | 2 | H | 2 | 32.19 | 29.41 |
| **LRRC37A** | 1 | 2 | H | 3 | 32.16 | 29.4 |
| **LRRC37A** | 1 | 2 | H | 4 | 31.71 | 29.31 |
| **LRRC37A** | 1 | 3 | G | 1 | 33.24 | 25.54 |
| **LRRC37A** | 1 | 3 | G | 2 | 33.48 | 25.57 |
| **LRRC37A** | 1 | 3 | G | 3 | 32.84 | 25.58 |
| **LRRC37A** | 1 | 3 | G | 4 | 32.68 | 25.39 |
| **LRRC37A** | 1 | 3 | H | 1 | 32.59 | 29.96 |
| **LRRC37A** | 1 | 3 | H | 2 | 32.68 | 29.63 |
| **LRRC37A** | 1 | 3 | H | 3 | 32.36 | 29.5 |
| **LRRC37A** | 1 | 3 | H | 4 | 32.23 | 29.37 |
| **LRRC37A** | 1 | 4 | G | 1 | 33.43 | 25.48 |
| **LRRC37A** | 1 | 4 | G | 2 | 32.89 | 25.71 |
| **LRRC37A** | 1 | 4 | G | 3 | 33.15 | 25.77 |
| **LRRC37A** | 1 | 4 | G | 4 | 33.34 | 25.51 |
| **LRRC37A** | 1 | 4 | H | 1 | 32.79 | 29.81 |
| **LRRC37A** | 1 | 4 | H | 2 | 33.16 | 30.2 |
| **LRRC37A** | 1 | 4 | H | 3 | 32.9 | 30.24 |
| **LRRC37A** | 1 | 4 | H | 4 | 32.6 | 29.83 |
| **LRRC37A** | 1 | 5 | G | 1 | 33.17 | 26.09 |
| **LRRC37A** | 1 | 5 | G | 2 | 33.02 | 26.01 |
| **LRRC37A** | 1 | 5 | G | 3 | 32.44 | 25.67 |
| **LRRC37A** | 1 | 5 | G | 4 | 32.52 | 25.73 |
| **LRRC37A** | 1 | 5 | H | 1 | 32.54 | 29.54 |
| **LRRC37A** | 1 | 5 | H | 2 | 32.41 | 29.45 |
| **LRRC37A** | 1 | 5 | H | 3 | 32.34 | 29.35 |
| **LRRC37A** | 1 | 5 | H | 4 | 32.32 | 29.27 |
| **LRRC37A2** | 1 | 1 | G | 1 | 32.62 | 25.47 |
| **LRRC37A2** | 1 | 1 | G | 2 | 32.86 | 25.43 |
| **LRRC37A2** | 1 | 1 | G | 3 | 32.69 | 25.47 |
| **LRRC37A2** | 1 | 1 | G | 4 | 32.52 | 25.43 |
| **LRRC37A2** | 1 | 1 | H | 1 | 32.11 | 29.08 |
| **LRRC37A2** | 1 | 1 | H | 2 | 32.31 | 28.94 |
| **LRRC37A2** | 1 | 1 | H | 3 | 32.19 | 29.05 |
| **LRRC37A2** | 1 | 1 | H | 4 | 32.46 | 29.07 |
| **LRRC37A2** | 1 | 2 | G | 1 | 33 | 25.64 |
| **LRRC37A2** | 1 | 2 | G | 2 | 32.92 | 25.53 |
| **LRRC37A2** | 1 | 2 | G | 3 | 32.92 | 25.49 |
| **LRRC37A2** | 1 | 2 | G | 4 | 32.74 | 25.49 |
| **LRRC37A2** | 1 | 2 | H | 1 | 32.87 | 29.44 |
| **LRRC37A2** | 1 | 2 | H | 2 | 32.48 | 29.21 |
| **LRRC37A2** | 1 | 2 | H | 3 | 32.81 | 29.53 |
| **LRRC37A2** | 1 | 2 | H | 4 | 32.57 | 29.2 |
| **LRRC37A2** | 1 | 3 | G | 1 | 33.91 | 25.47 |
| **LRRC37A2** | 1 | 3 | G | 2 | 33.4 | 25.53 |
| **LRRC37A2** | 1 | 3 | G | 3 | 33.51 | 25.37 |
| **LRRC37A2** | 1 | 3 | G | 4 | 34.04 | 25.4 |
| **LRRC37A2** | 1 | 3 | H | 1 | 33.65 | 29.51 |
| **LRRC37A2** | 1 | 3 | H | 2 | 33.41 | 29.58 |
| **LRRC37A2** | 1 | 3 | H | 3 | 33.62 | 29.55 |
| **LRRC37A2** | 1 | 3 | H | 4 | 33.56 | 29.57 |
| **LRRC37A2** | 1 | 4 | G | 1 | 34.32 | 26.12 |
| **LRRC37A2** | 1 | 4 | G | 2 | 34.48 | 26.08 |
| **LRRC37A2** | 1 | 4 | G | 3 | 34.47 | 25.89 |
| **LRRC37A2** | 1 | 4 | G | 4 | 34.15 | 25.71 |
| **LRRC37A2** | 1 | 4 | H | 1 | 33.72 | 29.92 |
| **LRRC37A2** | 1 | 4 | H | 2 | 34.16 | 30.04 |
| **LRRC37A2** | 1 | 4 | H | 3 | 34.28 | 30.22 |
| **LRRC37A2** | 1 | 4 | H | 4 | 34.2 | 30.18 |
| **LRRC37A2** | 1 | 5 | G | 1 | 33.29 | 26.13 |
| **LRRC37A2** | 1 | 5 | G | 2 | 33.19 | 26.05 |
| **LRRC37A2** | 1 | 5 | G | 3 | 33.05 | 26.09 |
| **LRRC37A2** | 1 | 5 | G | 4 | 33.3 | 26 |
| **LRRC37A2** | 1 | 5 | H | 1 | 33.1 | 29.67 |
| **LRRC37A2** | 1 | 5 | H | 2 | 33.09 | 29.44 |
| **LRRC37A2** | 1 | 5 | H | 3 | 32.95 | 29.42 |
| **LRRC37A2** | 1 | 5 | H | 4 | 33.05 | 29.53 |
| **NSF** | 1 | 1 | G | 1 | 30.24 | 26.11 |
| **NSF** | 1 | 1 | G | 2 | 30.35 | 26.09 |
| **NSF** | 1 | 1 | G | 3 | 30.34 | 26.09 |
| **NSF** | 1 | 1 | G | 4 | 30.3 | 26.12 |
| **NSF** | 1 | 1 | H | 1 | 31.57 | 29.48 |
| **NSF** | 1 | 1 | H | 2 | 31.68 | 29.45 |
| **NSF** | 1 | 1 | H | 3 | 31.91 | 29.43 |
| **NSF** | 1 | 1 | H | 4 | 31.62 | 29.47 |
| **NSF** | 1 | 2 | G | 1 | 30.51 | 26.32 |
| **NSF** | 1 | 2 | G | 2 | 30.68 | 26.26 |
| **NSF** | 1 | 2 | G | 3 | 30.66 | 26.21 |
| **NSF** | 1 | 2 | G | 4 | 30.68 | 26.18 |
| **NSF** | 1 | 2 | H | 1 | 31.91 | 29.63 |
| **NSF** | 1 | 2 | H | 2 | 31.96 | 29.53 |
| **NSF** | 1 | 2 | H | 3 | 31.97 | 29.25 |
| **NSF** | 1 | 2 | H | 4 | 31.81 | 29.23 |
| **NSF** | 1 | 3 | G | 1 | 31.05 | 26.2 |
| **NSF** | 1 | 3 | G | 2 | 30.66 | 26.08 |
| **NSF** | 1 | 3 | G | 3 | 30.8 | 25.9 |
| **NSF** | 1 | 3 | G | 4 | 31.06 | 26.13 |
| **NSF** | 1 | 3 | H | 1 | 32.2 | 30.01 |
| **NSF** | 1 | 3 | H | 2 | 32.22 | 30.03 |
| **NSF** | 1 | 3 | H | 3 | 32.35 | 30.12 |
| **NSF** | 1 | 3 | H | 4 | 32.43 | 30.06 |
| **NSF** | 1 | 4 | G | 1 | 31.26 | 26.61 |
| **NSF** | 1 | 4 | G | 2 | 31.2 | 26.45 |
| **NSF** | 1 | 4 | G | 3 | 31.14 | 26.3 |
| **NSF** | 1 | 4 | G | 4 | 31.04 | 26.28 |
| **NSF** | 1 | 4 | H | 1 | 32.48 | 30.55 |
| **NSF** | 1 | 4 | H | 2 | 32.26 | 30.14 |
| **NSF** | 1 | 4 | H | 3 | 32.36 | 30.1 |
| **NSF** | 1 | 4 | H | 4 | 32.51 | 30.06 |
| **NSF** | 1 | 5 | G | 1 | 31.24 | 26.85 |
| **NSF** | 1 | 5 | G | 2 | 30.96 | 26.62 |
| **NSF** | 1 | 5 | G | 3 | 31.03 | 26.73 |
| **NSF** | 1 | 5 | G | 4 | 31.29 | 26.7 |
| **NSF** | 1 | 5 | H | 1 | 32.07 | 29.88 |
| **NSF** | 1 | 5 | H | 2 | 31.97 | 29.88 |
| **NSF** | 1 | 5 | H | 3 | 32.06 | 29.73 |
| **NSF** | 1 | 5 | H | 4 | 32.06 | 30.07 |
| **MAPT** | 2 | 1 | G | 1 | 32.82 | 26.12 |
| **MAPT** | 2 | 1 | G | 2 | 32.89 | 26.14 |
| **MAPT** | 2 | 1 | G | 3 | 32.37 | 26.19 |
| **MAPT** | 2 | 1 | G | 4 | 32.84 | 26.22 |
| **MAPT** | 2 | 1 | H | 1 | 34.16 | 30.28 |
| **MAPT** | 2 | 1 | H | 2 | 34.18 | 30.14 |
| **MAPT** | 2 | 1 | H | 3 | 34.03 | 30.12 |
| **MAPT** | 2 | 1 | H | 4 | 33.99 | 30.27 |
| **MAPT** | 2 | 2 | G | 1 | 32.23 | 25.55 |
| **MAPT** | 2 | 2 | G | 2 | 32.26 | 25.66 |
| **MAPT** | 2 | 2 | G | 3 | 32.18 | 25.56 |
| **MAPT** | 2 | 2 | G | 4 | 32.14 | 25.63 |
| **MAPT** | 2 | 2 | H | 1 | 32.92 | 29.09 |
| **MAPT** | 2 | 2 | H | 2 | 33.35 | 29.03 |
| **MAPT** | 2 | 2 | H | 3 | 33.03 | 29.03 |
| **MAPT** | 2 | 2 | H | 4 | 32.94 | 29.1 |
| **MAPT** | 2 | 3 | G | 1 | 33.34 | 27.29 |
| **MAPT** | 2 | 3 | G | 2 | 33.23 | 27.19 |
| **MAPT** | 2 | 3 | G | 3 | 33.24 | 27.11 |
| **MAPT** | 2 | 3 | G | 4 | 32.83 | 27.08 |
| **MAPT** | 2 | 3 | H | 1 | 34.77 | 31.41 |
| **MAPT** | 2 | 3 | H | 2 | 34.95 | 31.39 |
| **MAPT** | 2 | 3 | H | 3 | 34.56 | 31.5 |
| **MAPT** | 2 | 3 | H | 4 | 35.05 | 30.79 |
| **MAPT** | 2 | 4 | G | 1 | 33.27 | 27.34 |
| **MAPT** | 2 | 4 | G | 2 | 33.91 | 27.49 |
| **MAPT** | 2 | 4 | G | 3 | 34.06 | 27.56 |
| **MAPT** | 2 | 4 | G | 4 | 33.93 | 27.53 |
| **MAPT** | 2 | 4 | H | 1 | 35.37 | 31.13 |
| **MAPT** | 2 | 4 | H | 2 | 34.81 | 30.96 |
| **MAPT** | 2 | 4 | H | 3 | 35.24 | 31.03 |
| **MAPT** | 2 | 4 | H | 4 | 35.42 | 30.8 |
| **MAPT** | 2 | 5 | G | 1 | 33.75 | 27.74 |
| **MAPT** | 2 | 5 | G | 2 | 34.1 | 27.84 |
| **MAPT** | 2 | 5 | G | 3 | 34.34 | 27.82 |
| **MAPT** | 2 | 5 | G | 4 | 33.53 | 27.68 |
| **MAPT** | 2 | 5 | H | 1 | 35.11 | 30.89 |
| **MAPT** | 2 | 5 | H | 2 | 35.23 | 31.3 |
| **MAPT** | 2 | 5 | H | 3 | 34.46 | 31.33 |
| **MAPT** | 2 | 5 | H | 4 | 35.02 | 31.09 |
| **KANSL1** | 2 | 1 | G | 1 | 31.49 | 24.07 |
| **KANSL1** | 2 | 1 | G | 2 |  |  |
| **KANSL1** | 2 | 1 | G | 3 | 31.62 | 24.02 |
| **KANSL1** | 2 | 1 | G | 4 | 32.05 | 24.12 |
| **KANSL1** | 2 | 1 | H | 1 | 32.6 | 32.31 |
| **KANSL1** | 2 | 1 | H | 2 | 32.67 | 32.66 |
| **KANSL1** | 2 | 1 | H | 3 | 32.44 | 32.62 |
| **KANSL1** | 2 | 1 | H | 4 | 32.43 | 32.15 |
| **KANSL1** | 2 | 2 | G | 1 | 30.16 | 22.9 |
| **KANSL1** | 2 | 2 | G | 2 | 30.16 | 22.79 |
| **KANSL1** | 2 | 2 | G | 3 | 30.16 | 22.88 |
| **KANSL1** | 2 | 2 | G | 4 | 30.25 | 22.9 |
| **KANSL1** | 2 | 2 | H | 1 | 30.41 | 29.88 |
| **KANSL1** | 2 | 2 | H | 2 | 30.44 | 29.86 |
| **KANSL1** | 2 | 2 | H | 3 | 30.45 | 29.67 |
| **KANSL1** | 2 | 2 | H | 4 | 30.52 | 29.99 |
| **KANSL1** | 2 | 3 | G | 1 | 30.22 | 22.12 |
| **KANSL1** | 2 | 3 | G | 2 | 30.29 | 22.11 |
| **KANSL1** | 2 | 3 | G | 3 | 30.14 | 22.08 |
| **KANSL1** | 2 | 3 | G | 4 | 30.1 | 22.06 |
| **KANSL1** | 2 | 3 | H | 1 | 30.61 | 29.56 |
| **KANSL1** | 2 | 3 | H | 2 | 30.28 | 29.45 |
| **KANSL1** | 2 | 3 | H | 3 | 30.33 | 29.24 |
| **KANSL1** | 2 | 3 | H | 4 | 30.36 | 29.66 |
| **KANSL1** | 2 | 4 | G | 1 | 30.59 | 22.06 |
| **KANSL1** | 2 | 4 | G | 2 | 30.51 | 22.4 |
| **KANSL1** | 2 | 4 | G | 3 | 30.45 | 22.29 |
| **KANSL1** | 2 | 4 | G | 4 | 30.46 | 22.34 |
| **KANSL1** | 2 | 4 | H | 1 | 30.84 | 29.74 |
| **KANSL1** | 2 | 4 | H | 2 | 30.73 | 29.62 |
| **KANSL1** | 2 | 4 | H | 3 | 30.75 | 30.04 |
| **KANSL1** | 2 | 4 | H | 4 | 30.95 | 29.75 |
| **KANSL1** | 2 | 5 | G | 1 | 31.58 | 23.38 |
| **KANSL1** | 2 | 5 | G | 2 | 31.45 | 23.25 |
| **KANSL1** | 2 | 5 | G | 3 | 31.55 | 23.34 |
| **KANSL1** | 2 | 5 | G | 4 | 31.5 | 23.47 |
| **KANSL1** | 2 | 5 | H | 1 | 31.38 | 31.04 |
| **KANSL1** | 2 | 5 | H | 2 | 31.62 | 30.52 |
| **KANSL1** | 2 | 5 | H | 3 | 31.7 | 31.04 |
| **KANSL1** | 2 | 5 | H | 4 | 31.53 | 30.53 |
| **ARL17A** | 2 | 1 | G | 1 | 34.54 | 23.05 |
| **ARL17A** | 2 | 1 | G | 2 | 34.64 | 23.01 |
| **ARL17A** | 2 | 1 | G | 3 | 35.19 | 23.02 |
| **ARL17A** | 2 | 1 | G | 4 | 35.12 | 23.03 |
| **ARL17A** | 2 | 1 | H | 1 | 34.25 | 30.68 |
| **ARL17A** | 2 | 1 | H | 2 | 34.61 | 30.87 |
| **ARL17A** | 2 | 1 | H | 3 | 34 | 30.88 |
| **ARL17A** | 2 | 1 | H | 4 | 34.32 | 30.77 |
| **ARL17A** | 2 | 2 | G | 1 | 33.92 | 22.88 |
| **ARL17A** | 2 | 2 | G | 2 | 34.77 | 22.8 |
| **ARL17A** | 2 | 2 | G | 3 | 34.21 | 22.75 |
| **ARL17A** | 2 | 2 | G | 4 | 34.35 | 22.75 |
| **ARL17A** | 2 | 2 | H | 1 | 33.61 | 29.54 |
| **ARL17A** | 2 | 2 | H | 2 | 33.23 | 29.64 |
| **ARL17A** | 2 | 2 | H | 3 | 34.14 | 29.32 |
| **ARL17A** | 2 | 2 | H | 4 | 34.06 | 29.36 |
| **ARL17A** | 2 | 3 | G | 1 | 35 | 22.52 |
| **ARL17A** | 2 | 3 | G | 2 | 35.01 | 22.1 |
| **ARL17A** | 2 | 3 | G | 3 | 35.03 | 22.04 |
| **ARL17A** | 2 | 3 | G | 4 | 34.57 | 22.04 |
| **ARL17A** | 2 | 3 | H | 1 | 36.49 | 32.62 |
| **ARL17A** | 2 | 3 | H | 2 | 37.22 | 32.59 |
| **ARL17A** | 2 | 3 | H | 3 | 35.43 | 32.59 |
| **ARL17A** | 2 | 3 | H | 4 | 37.09 | 32.53 |
| **ARL17A** | 2 | 4 | G | 1 | 34.28 | 22.29 |
| **ARL17A** | 2 | 4 | G | 2 | 35.15 | 22.31 |
| **ARL17A** | 2 | 4 | G | 3 | 34.08 | 22.42 |
| **ARL17A** | 2 | 4 | G | 4 | 35.11 | 22.31 |
| **ARL17A** | 2 | 4 | H | 1 | 37.06 | 31.89 |
| **ARL17A** | 2 | 4 | H | 2 | 36.31 | 32.31 |
| **ARL17A** | 2 | 4 | H | 3 | 38.12 | 31.87 |
| **ARL17A** | 2 | 4 | H | 4 | 37.34 | 32.03 |
| **ARL17A** | 2 | 5 | G | 1 | 35.03 | 22.95 |
| **ARL17A** | 2 | 5 | G | 2 | 36.2 | 23.14 |
| **ARL17A** | 2 | 5 | G | 3 | 34.64 | 23.16 |
| **ARL17A** | 2 | 5 | G | 4 | 35.28 | 23.25 |
| **ARL17A** | 2 | 5 | H | 1 | 35.45 | 31.17 |
| **ARL17A** | 2 | 5 | H | 2 | 35.42 | 32.02 |
| **ARL17A** | 2 | 5 | H | 3 | 35.59 | 31.3 |
| **ARL17A** | 2 | 5 | H | 4 | 38.11 | 31.47 |
| **ARL17B** | 2 | 1 | G | 1 | 34.4 | 27.4 |
| **ARL17B** | 2 | 1 | G | 2 | 35.54 | 27.41 |
| **ARL17B** | 2 | 1 | G | 3 | 34.18 | 27.39 |
| **ARL17B** | 2 | 1 | G | 4 | 34.98 | 27.29 |
| **ARL17B** | 2 | 1 | H | 1 | 35.32 | 30.07 |
| **ARL17B** | 2 | 1 | H | 2 | 35.06 | 29.9 |
| **ARL17B** | 2 | 1 | H | 3 | 35.95 | 30.87 |
| **ARL17B** | 2 | 1 | H | 4 | 36.04 | 30.03 |
| **ARL17B** | 2 | 2 | G | 1 | 33.43 | 26.52 |
| **ARL17B** | 2 | 2 | G | 2 | 33.33 | 26.62 |
| **ARL17B** | 2 | 2 | G | 3 | 33.24 | 26.41 |
| **ARL17B** | 2 | 2 | G | 4 | 33.35 | 26.65 |
| **ARL17B** | 2 | 2 | H | 1 | 35.08 | 29.07 |
| **ARL17B** | 2 | 2 | H | 2 | 34.32 | 29.24 |
| **ARL17B** | 2 | 2 | H | 3 | 34.59 | 29.07 |
| **ARL17B** | 2 | 2 | H | 4 | 34.2 | 29.78 |
| **ARL17B** | 2 | 3 | G | 1 | 37.27 | 28.09 |
| **ARL17B** | 2 | 3 | G | 2 | 26.11 | 28.1 |
| **ARL17B** | 2 | 3 | G | 3 | 35.91 | 28.33 |
| **ARL17B** | 2 | 3 | G | 4 | 36.7 | 28.09 |
| **ARL17B** | 2 | 3 | H | 1 | 37.47 | 30.79 |
| **ARL17B** | 2 | 3 | H | 2 | 38.52 | 30.43 |
| **ARL17B** | 2 | 3 | H | 3 | 37.26 | 30.63 |
| **ARL17B** | 2 | 3 | H | 4 | 27.42 | 30.54 |
| **ARL17B** | 2 | 4 | G | 1 | 35.33 | 28.33 |
| **ARL17B** | 2 | 4 | G | 2 | 35.18 | 28.38 |
| **ARL17B** | 2 | 4 | G | 3 | 35.27 | 28.45 |
| **ARL17B** | 2 | 4 | G | 4 | 37.11 | 28.27 |
| **ARL17B** | 2 | 4 | H | 1 | 36.29 | 30.55 |
| **ARL17B** | 2 | 4 | H | 2 | 37.03 | 30.73 |
| **ARL17B** | 2 | 4 | H | 3 | 37.26 | 30.27 |
| **ARL17B** | 2 | 4 | H | 4 | 37.51 | 30.52 |
| **ARL17B** | 2 | 5 | G | 1 | 35.64 | 28.82 |
| **ARL17B** | 2 | 5 | G | 2 | 36.35 | 28.81 |
| **ARL17B** | 2 | 5 | G | 3 | 36.08 | 29.04 |
| **ARL17B** | 2 | 5 | G | 4 | 35.45 | 28.89 |
| **ARL17B** | 2 | 5 | H | 1 | 36.82 | 30.73 |
| **ARL17B** | 2 | 5 | H | 2 | 36.81 | 31.02 |
| **ARL17B** | 2 | 5 | H | 3 | 36.77 | 30.89 |
| **ARL17B** | 2 | 5 | H | 4 | 38.6 | 31.08 |
| **LRRC37A** | 2 | 1 | G | 1 | 33.69 | 24.22 |
| **LRRC37A** | 2 | 1 | G | 2 | 34.28 | 24.21 |
| **LRRC37A** | 2 | 1 | G | 3 | 34.13 | 24.22 |
| **LRRC37A** | 2 | 1 | G | 4 | 34.25 | 24.39 |
| **LRRC37A** | 2 | 1 | H | 1 | 32.74 | 30.14 |
| **LRRC37A** | 2 | 1 | H | 2 | 32.51 | 30.29 |
| **LRRC37A** | 2 | 1 | H | 3 | 32.52 | 30.19 |
| **LRRC37A** | 2 | 1 | H | 4 | 32.68 | 30.08 |
| **LRRC37A** | 2 | 2 | G | 1 | 32.06 | 22.78 |
| **LRRC37A** | 2 | 2 | G | 2 | 31.96 | 22.72 |
| **LRRC37A** | 2 | 2 | G | 3 | 32.08 | 22.85 |
| **LRRC37A** | 2 | 2 | G | 4 | 32.09 | 22.76 |
| **LRRC37A** | 2 | 2 | H | 1 | 31.54 | 29.23 |
| **LRRC37A** | 2 | 2 | H | 2 | 32.57 | 29.06 |
| **LRRC37A** | 2 | 2 | H | 3 | 31.92 | 29.13 |
| **LRRC37A** | 2 | 2 | H | 4 | 32.05 | 28.93 |
| **LRRC37A** | 2 | 3 | G | 1 | 32.09 | 21.65 |
| **LRRC37A** | 2 | 3 | G | 2 | 32.05 | 21.65 |
| **LRRC37A** | 2 | 3 | G | 3 | 32.17 | 21.46 |
| **LRRC37A** | 2 | 3 | G | 4 | 32.3 | 21.84 |
| **LRRC37A** | 2 | 3 | H | 1 | 33.61 | 30.93 |
| **LRRC37A** | 2 | 3 | H | 2 | 34.31 | 30.78 |
| **LRRC37A** | 2 | 3 | H | 3 | 32.92 | 30.9 |
| **LRRC37A** | 2 | 3 | H | 4 | 34.15 | 30.98 |
| **LRRC37A** | 2 | 4 | G | 1 | 32.15 | 22.23 |
| **LRRC37A** | 2 | 4 | G | 2 | 32.31 | 22.33 |
| **LRRC37A** | 2 | 4 | G | 3 | 32.58 | 22.05 |
| **LRRC37A** | 2 | 4 | G | 4 | 32.6 | 22.21 |
| **LRRC37A** | 2 | 4 | H | 1 | 34.35 | 31.49 |
| **LRRC37A** | 2 | 4 | H | 2 | 34.03 | 30.67 |
| **LRRC37A** | 2 | 4 | H | 3 | 33.75 | 31.4 |
| **LRRC37A** | 2 | 4 | H | 4 | 34.65 | 30.98 |
| **LRRC37A** | 2 | 5 | G | 1 | 34.22 | 23.04 |
| **LRRC37A** | 2 | 5 | G | 2 | 33.89 | 23.07 |
| **LRRC37A** | 2 | 5 | G | 3 | 33.93 | 23.14 |
| **LRRC37A** | 2 | 5 | G | 4 | 34.28 | 23.12 |
| **LRRC37A** | 2 | 5 | H | 1 | 33.69 | 31.04 |
| **LRRC37A** | 2 | 5 | H | 2 | 33.71 | 30.82 |
| **LRRC37A** | 2 | 5 | H | 3 | 34.11 | 31.12 |
| **LRRC37A** | 2 | 5 | H | 4 | 33.39 | 30.87 |
| **LRRC37A2** | 2 | 1 | G | 1 | 35.05 | 24.55 |
| **LRRC37A2** | 2 | 1 | G | 2 | 34.8 | 24.38 |
| **LRRC37A2** | 2 | 1 | G | 3 | 34.76 | 24.37 |
| **LRRC37A2** | 2 | 1 | G | 4 | 35.16 | 24.41 |
| **LRRC37A2** | 2 | 1 | H | 1 | 34.36 | 31.16 |
| **LRRC37A2** | 2 | 1 | H | 2 | 34.38 | 31.15 |
| **LRRC37A2** | 2 | 1 | H | 3 | 34.58 | 30.87 |
| **LRRC37A2** | 2 | 1 | H | 4 | 34.55 | 30.89 |
| **LRRC37A2** | 2 | 2 | G | 1 | 32.76 | 22.81 |
| **LRRC37A2** | 2 | 2 | G | 2 | 32.56 | 22.78 |
| **LRRC37A2** | 2 | 2 | G | 3 | 32.99 | 22.57 |
| **LRRC37A2** | 2 | 2 | G | 4 | 32.86 | 22.69 |
| **LRRC37A2** | 2 | 2 | H | 1 | 33.3 | 29.5 |
| **LRRC37A2** | 2 | 2 | H | 2 | 33.44 | 29.54 |
| **LRRC37A2** | 2 | 2 | H | 3 | 33.41 | 29.56 |
| **LRRC37A2** | 2 | 2 | H | 4 | 33.38 | 29.66 |
| **LRRC37A2** | 2 | 3 | G | 1 | 33.12 | 21.91 |
| **LRRC37A2** | 2 | 3 | G | 2 | 33.63 | 21.91 |
| **LRRC37A2** | 2 | 3 | G | 3 | 33.3 | 21.82 |
| **LRRC37A2** | 2 | 3 | G | 4 | 33.74 | 21.99 |
| **LRRC37A2** | 2 | 3 | H | 1 | 33.83 | 29.4 |
| **LRRC37A2** | 2 | 3 | H | 2 | 33.69 | 29.53 |
| **LRRC37A2** | 2 | 3 | H | 3 | 33.81 | 29.67 |
| **LRRC37A2** | 2 | 3 | H | 4 | 33.82 | 29.62 |
| **LRRC37A2** | 2 | 4 | G | 1 | 33.29 | 22.4 |
| **LRRC37A2** | 2 | 4 | G | 2 | 32.9 | 22.13 |
| **LRRC37A2** | 2 | 4 | G | 3 | 33.09 | 22.6 |
| **LRRC37A2** | 2 | 4 | G | 4 | 32.66 | 22.25 |
| **LRRC37A2** | 2 | 4 | H | 1 | 33.18 | 30.02 |
| **LRRC37A2** | 2 | 4 | H | 2 | 33.32 | 29.79 |
| **LRRC37A2** | 2 | 4 | H | 3 | 33.38 | 29.37 |
| **LRRC37A2** | 2 | 4 | H | 4 | 33.25 | 29.76 |
| **LRRC37A2** | 2 | 5 | G | 1 | 33.93 | 23.21 |
| **LRRC37A2** | 2 | 5 | G | 2 | 33.92 | 23.05 |
| **LRRC37A2** | 2 | 5 | G | 3 | 33.55 | 23.07 |
| **LRRC37A2** | 2 | 5 | G | 4 | 34.03 | 23.13 |
| **LRRC37A2** | 2 | 5 | H | 1 | 33.69 | 31.21 |
| **LRRC37A2** | 2 | 5 | H | 2 | 34.44 | 31.01 |
| **LRRC37A2** | 2 | 5 | H | 3 | 34.15 | 31.12 |
| **LRRC37A2** | 2 | 5 | H | 4 | 34.02 | 31 |
| **NSF** | 2 | 1 | G | 1 | 32.93 | 23.14 |
| **NSF** | 2 | 1 | G | 2 | 32.76 | 23.07 |
| **NSF** | 2 | 1 | G | 3 | 32.85 | 23.13 |
| **NSF** | 2 | 1 | G | 4 | 32.65 | 23.12 |
| **NSF** | 2 | 1 | H | 1 | 32.13 | 29.95 |
| **NSF** | 2 | 1 | H | 2 | 32.08 | 29.81 |
| **NSF** | 2 | 1 | H | 3 | 32.29 | 30.11 |
| **NSF** | 2 | 1 | H | 4 | 32.5 | 30.1 |
| **NSF** | 2 | 2 | G | 1 | 32.92 | 23.28 |
| **NSF** | 2 | 2 | G | 2 | 32.69 | 23.24 |
| **NSF** | 2 | 2 | G | 3 | 32.93 | 23.16 |
| **NSF** | 2 | 2 | G | 4 | 32.73 | 23.22 |
| **NSF** | 2 | 2 | H | 1 | 31.4 | 28.81 |
| **NSF** | 2 | 2 | H | 2 | 31.3 | 29 |
| **NSF** | 2 | 2 | H | 3 | 31.45 | 28.64 |
| **NSF** | 2 | 2 | H | 4 | 31.81 | 28.9 |
| **NSF** | 2 | 3 | G | 1 | 31.9 | 22.18 |
| **NSF** | 2 | 3 | G | 2 | 32.21 | 22.2 |
| **NSF** | 2 | 3 | G | 3 | 31.98 | 22.07 |
| **NSF** | 2 | 3 | G | 4 | 31.94 | 22.12 |
| **NSF** | 2 | 3 | H | 1 | 32.51 | 30.62 |
| **NSF** | 2 | 3 | H | 2 | 32.74 | 30.85 |
| **NSF** | 2 | 3 | H | 3 | 32.69 | 31.12 |
| **NSF** | 2 | 3 | H | 4 | 32.88 | 31.21 |
| **NSF** | 2 | 4 | G | 1 | 32.19 | 22.57 |
| **NSF** | 2 | 4 | G | 2 | 32.04 | 22.49 |
| **NSF** | 2 | 4 | G | 3 | 31.99 | 22.33 |
| **NSF** | 2 | 4 | G | 4 | 32.03 | 22.36 |
| **NSF** | 2 | 4 | H | 1 | 33 | 30.62 |
| **NSF** | 2 | 4 | H | 2 | 33.42 | 31.68 |
| **NSF** | 2 | 4 | H | 3 | 33.19 | 30.76 |
| **NSF** | 2 | 4 | H | 4 | 32.8 | 30.76 |
| **NSF** | 2 | 5 | G | 1 | 33.22 | 23.72 |
| **NSF** | 2 | 5 | G | 2 | 33.19 | 23.67 |
| **NSF** | 2 | 5 | G | 3 | 33.29 | 23.54 |
| **NSF** | 2 | 5 | G | 4 | 33.25 | 23.53 |
| **NSF** | 2 | 5 | H | 1 | 33.14 | 31 |
| **NSF** | 2 | 5 | H | 2 | 32.95 | 30.52 |
| **NSF** | 2 | 5 | H | 3 | 32.84 | 30.47 |
| **NSF** | 2 | 5 | H | 4 | 32.92 | 30.8 |
